# HALD, a human aging and longevity knowledge graph for precision gerontology and geroscience analyses

**DOI:** 10.1038/s41597-023-02781-0

**Published:** 2023-12-01

**Authors:** Zexu Wu, Cong Feng, Yanshi Hu, Yincong Zhou, Sida Li, Shilong Zhang, Yueming Hu, Yuhao Chen, Haoyu Chao, Qingyang Ni, Ming Chen

**Affiliations:** 1 Department of Bioinformatics, College of Life Sciences, Zhejiang University, Hangzhou, 310058 China; 2 The First Affiliated Hospital, Zhejiang University School of Medicine; Institute of Hematology, Zhejiang University, Hangzhou, 310058 China; 3 Joint Research Centre for Engineering Biology, Zhejiang University-University of Edinburgh Institute, Zhejiang University, Haining, 314400 China

**Keywords:** Data integration, Literature mining, Bioinformatics, Biochemical reaction networks, Data mining

## Abstract

Human aging is a natural and inevitable biological process that leads to an increased risk of aging-related diseases. Developing anti-aging therapies for aging-related diseases requires a comprehensive understanding of the mechanisms and effects of aging and longevity from a multi-modal and multi-faceted perspective. However, most of the relevant knowledge is scattered in the biomedical literature, the volume of which reached 36 million in PubMed. Here, we presented HALD, a text mining-based human aging and longevity dataset of the biomedical knowledge graph from all published literature related to human aging and longevity in PubMed. HALD integrated multiple state-of-the-art natural language processing (NLP) techniques to improve the accuracy and coverage of the knowledge graph for precision gerontology and geroscience analyses. Up to September 2023, HALD had contained 12,227 entities in 10 types (gene, RNA, protein, carbohydrate, lipid, peptide, pharmaceutical preparations, toxin, mutation, and disease), 115,522 relations, 1,855 aging biomarkers, and 525 longevity biomarkers from 339,918 biomedical articles in PubMed. HALD is available at https://bis.zju.edu.cn/hald.

## Background & Summary

Aging is a natural biological process accompanied by a gradual decline of physiological functions and associated with an increased risk of aging-related diseases^1^. Longevity refers to the extension of an individual’s lifespan and the sustained maintenance of their health status. Research into human aging and longevity aims to explore the mechanisms of human health and life, with the hope of discovering approaches to slow down aging and increase lifespan. Human aging and longevity are mainly influenced by a wide range of genetic, epigenetic, and environmental factors^2^. As the global population continues to age, the study of aging and longevity at the molecular level has become a hot topic in the field of gerontology and geroscience. Precision gerontology aims to provide predictions regarding the individuals’ lifespan under various treatment scenarios^3^, while the main objective of geroscience is to devise novel, biologically-driven therapeutic and preventive strategies that address fundamental aging mechanisms^4^. Understanding the molecular basis of human aging and longevity is vital for the development of therapies to prevent aging-related diseases and extend healthspan and lifespan.

The published literature is one of the most accessible data sources of molecular and disease information related to aging and longevity. However, due to the huge amount of biomedical literature, it is time-consuming and inefficient for researchers to conduct information retrieval from the major databases of medical journals like PubMed. In recent years, deep learning (DL) models have achieved great success for automated extraction in named entity recognition and relation extraction from the biomedical literature^5^. Besides, several web-based applications for automated text mining such as PubTator^6^ and pubmedKB^7^ provide information about biomedical entities including gene, disease, chemical, mutation, species, and cell line from all published biomedical literature.

Integrated datasets with comprehensive knowledge are crucial for researchers to leverage existing resources. Currently, there are some publicly online manually curated databases related to human aging and longevity, such as Aging genes/interventions database (AGEID)^8^, Human Ageing Genomic Resources (HAGR)^9^, JenAge Ageing Factor Database (AgeFactDB)^10^, Aging Atlas^11^, and AgingBank^12^ (Table 1). AGEID is a database of experimental results that provides formatted gene/intervention reports related to aging^8^. HAGR includes the GenAge, AnAge, GenDR, LongevityMap, DrugAge and CellAge databases that are manually curated by experts and regularly updated^9^. AgeFactDB is aimed at the collection and integration of aging-related data including genes, chemical compounds, and other environmental cues^10^. Aging Atlas is a manually curated biomedical database comprising a range of aging-related multi-omics datasets and bioinformatics tools^11^. AgingBank documents high-quality aging-related associations in more than 50 species by manually reviewing more than 20,000 publicly published papers^12^. However, to the best of our knowledge, these databases are all manually curated, making it difficult to incorporate comprehensive knowledge of human aging and longevity. It is also difficult to obtain the latest biomedical knowledge from manually curated databases as their services are out of maintenance or not updated in time. In addition, although human nucleic acids information is generally involved in these studies, knowledge of other important organic compounds like carbohydrate, lipid, and protein is not yet fully integrated. Relation extraction between these entities is also indispensable for researchers to facilitate integrative and comprehensive analysis. Associations between molecular markers and diseases also must be clarified to illuminate the mechanisms and effects of anti-aging therapies on aging-related diseases^13^.

**Table 1:** Summary of human aging and longevity-related databases.

Databases	Aging/Longevity	Data	Last Update*
AGEID (2002)^8^	Aging and longevity	Genes and interventions	Not available
AnAge (2013)^9^	Aging and longevity	Aging and life history	Build 15 (July 3, 2023)
GenAge (2013)^9^	Aging	Genes	Build 21 (August 28, 2023)
LongevityMap (2013)^9^	Longevity	Genetic variants	Build 3 (June 24, 2023)
AgeFactDB (2014)^10^	Aging	Genes, chemical compounds and other environmental cues	Not available
Aging Atlas (2020)^11^	Aging	Multi-omics datasets	January 10, 2023

*Note: The accessed date for all databases is October 26, 2023.

A knowledge graph (KG) is widely used for knowledge domain visualization or knowledge domain mapping graphs in the library and information industry^14^. In the field of life sciences, a biomedical KG can not only link biomedical entities through certain relations, but also predict the potential relationships between existing entities and discover new relational facts^15^. Such characteristics can facilitate the understanding of relations between biomedical entities, which is crucial for researchers to refine their research scope.

**Fig. 1:**
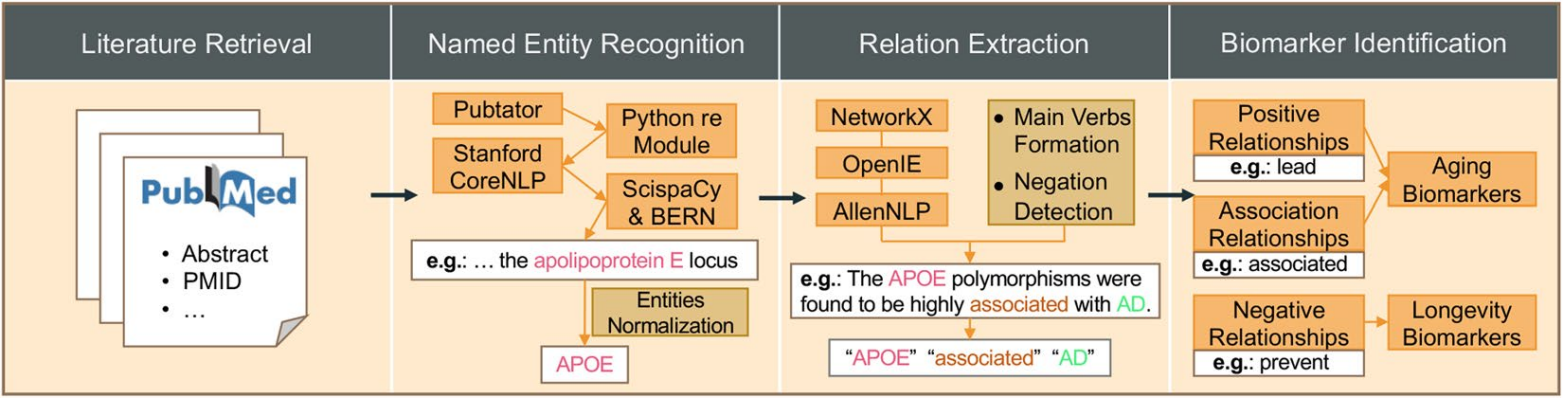
The workflow of HALD. (1) In the Literature Retrieval phase, we collected abstracts, PMIDs, and other information from PubMed. (2) In the Named Entity Recognition phase, we employed PubTator, Python’s re Module, Stanford CoreNLP, ScispaCy, and BERN methods to identify and normalize named entities. (3) In the Relation Extraction phase, we used NetworkX, OpenIE, and AllenNLP tools to extract relations, in which Main Verbs Formation and Negation Detection were included. (4) In the Biomarkers Identification phase, we classified the relationships into positive, association, and negative ones based on their types. Further identification as biomarkers for human aging and longevity was performed.

**Fig. 2:**
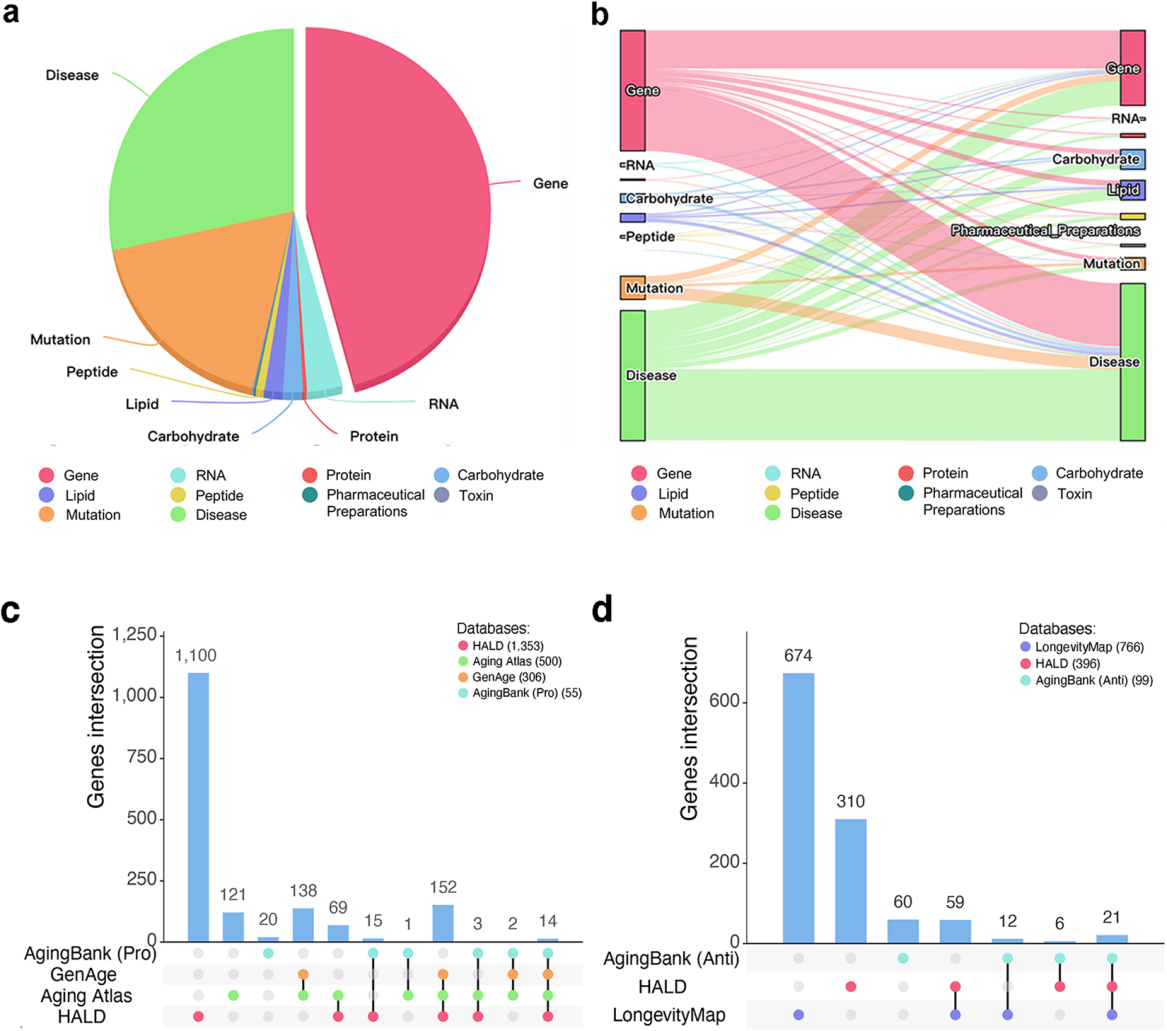
The distribution and evaluation of HALD. (**a**) The pie chart of entity distribution. (**b**) The Sankey diagram of relation distribution. (**c**) The comparison of aging-related gene counts among HALD, Aging Atlas, GenAge and AgingBank (Pro). (**d**) The comparison of longevity-related gene counts among LongevityMap, HALD, and AgingBank (Pro).

In this paper, we presented HALD, a human aging and longevity dataset of the biomedical KG from human aging and longevity-related literature in PubMed. Figure 1 illustrates the workflow of biomedical literature mining using multiple NLP techniques. First, we used the Bio.Entrez^16^ python package to conduct literature retrieval. Then, we took web-based (PubTator^6^), dictionary-based (Python re module), rule-based (Stanford CoreNLP^17^), and DL-based (ScispaCy^18^ and BERN^19^) methods to conduct named entity recognition (NER) for better accuracy. Next, we combined NetworkX, OpenIE, and AllenNLP tools to conduct relation extraction (RE) for wider coverage. Finally, the entities were further identified as human aging and longevity biomarkers according to their relationships with aging-related diseases. Up to September 2023, we had annotated 339,918 abstracts from PubMed and curated 12,227 entities in 10 types (gene, RNA, carbohydrate, peptide, lipid, protein, pharmaceutical preparations, toxin, mutation, and disease entities), 115,522 relations, 1,855 aging biomarkers, and 525 longevity biomarkers in HALD. The distributions of entities and relations are shown in Fig. 2a,b. The contributions of HALD are listed as followings:

- HALD is the first human aging and longevity knowledge dataset of the biomedical knowledge graph mined from published literature using NLP technologies.
- HALD provides 10 types of credible human aging and longevity biomedical entities.
- HALD links biomedical entities through certain relations and predicts the potential relationships.
- HALD identifies aging and longevity biomarkers from curated entities and elucidates their associations with aging-related diseases.

## Methods

### Literature retrieval

A search for (“aging” [Title/Abstract] OR “ageing” [Title/Abstract] OR “longevity” [Title/Abstract] OR “centenarian” [Title/Abstract] OR “the elderly” [Title/Abstract] OR “the aged” [Title/Abstract] OR “old people” [Title/Abstract] OR “older people” [Title/Abstract] OR “old age” [Title/Abstract] OR “gerontology” [Title/Abstract] OR “geroscience” [Title/Abstract] OR “lifespan” [Title/Abstract] OR “healthspan” [Title/Abstract] OR “life expectancy” [Title/Abstract] AND “Journal Article” [ptyp] AND “humans” [MeSH Terms] AND “English” [lang]) was used to retrieve PubMed biomedical journal articles related to human aging and longevity directly with the Bio.Entrez python package. Bio.Entrez python package can translate a standard set of input parameters into the values necessary for various National Center for Biotechnology Information (NCBI) software components to search for and retrieve the requested data^16^. In addition, we retrieved the full names, abbreviations, journal impact factors (IF), and five-year journal impact factors (IF5) of these journals from JCR (https://jcr.clarivate.com). JCR provides various indicators including IF, which is mainly used to evaluate the influence and academic quality of journals. The magnitude of IF is considered one of the important metrics for measuring a journal’s influence in the academic community.

### Named entity recognition

We combined web-based, dictionary-based, rule-based, and DL-based methods to conduct NER, and recognized 10 types of entities including gene, RNA, protein, carbohydrate, lipid, peptide, pharmaceutical preparations, toxin, mutation, and disease.

#### Web-based method

PubMed unique identifiers (PMIDs) were submitted to the web-based method PubTator to get the annotations of 5 types of entities (gene, disease, chemical, mutation, and species). PubTator provides state-of-the-art performance on generating automatic computer pre-annotations in computer-assisted biocuration^6^. Multiple text-mining tools are integrated in PubTator, including GNormPlus for identifying gene/protein entities^20^, TmVar for identifying gene variants^21^, and DNorm for identifying disease entities^22^. PubTator is updated with new PubMed articles daily, which is a great library for biomedical literature text mining.

#### Dictionary-based method

We used Python re module to conduct the dictionary-based method NER by matching words or phrases in the sentences with the items in human gene, RNA, carbohydrate, peptide, lipid, protein, pharmaceutical preparations, toxin, mutation, and disease dictionaries. HUGO Gene Nomenclature Committee (HGNC) is responsible for approving unique symbols and names for human loci, including protein-coding genes and non-coding RNAs^23^. Medical subject headings (MeSH) is a comprehensive controlled vocabulary for indexing journal articles and books in the life sciences^24^. Each MeSH record consists of one or more concepts, and each concept consists in one or more synonymous terms. The single nucleotide polymorphism database (dbSNP) is a public database within NCBI that documents single nucleotide polymorphisms and other types of genetic variation in the human species^25^. Therefore, we downloaded and pre-processed the files of genes and RNA in HGNC as the standard human gene and RNA dictionaries, including the information about approved symbol, approved name, previous symbols, alias symbols, previous name, alias names, and NCBI gene ID. We queried the concepts and terms of biochemical molecules in MeSH, and collated the entry information of the following six categories as dictionaries: carbohydrate, peptide, lipid, protein, pharmaceutical preparations, and toxin. Similarly, we built disease dictionaries according to disease heading in MeSH. We collected mutation information about alleles and position in dbSNP and organized them into mutation dictionaries. Subsequently, we used the regular expression matching operations provided by Python re module (https://docs.python.org/3/library/re.html) to match these ten types of entities in each sentence annotated by Pubtator according to the dictionaries. The entities that did not match the dictionaries would not be retained.

#### Rule-based method

The rule-based method was added using regular expression patterns to match rules for miRNA and lncRNA entities as a complement to the RNA entities. miRNA and lncRNA entities have relatively fixed naming rules. For instance, miRNA entities always begin with “MIR”, “microRNA”, “MIRN”, and “hsa-miR”, while lncRNA entities usually begin with “linc”. Stanford CoreNLP, a set of natural language analysis tools written in Java^17^, was applied to RNA entity recognition through the rule-based method.

#### DL-based method

The DL-based methods ScispaCy and BERN were employed for more accurate recognition of gene entities. ScispaCy, a specialized NLP library for processing biomedical texts building on the robust spaCy^26^ library, provides the models of the JNLPA corpus (F1-score = 72.28%), the BC5CDR corpus (F1-score = 84.53%) and the BIONLP13CG corpus (F1-score = 76.57%) for gene entities recognition^18^. Besides, BERN, a biomedical text mining tool that uses neural network-based high-performance BioBERT^27^ NER models for recognizing known entities and discovering new entities^19^, was also used to recognize gene entities.

### Relation extraction

Once two entities co-exist in one sentence, and a main verb lies between the 2 entities at the meanwhile, there is likely to be some relationship between these two entities. We selected sentences with no less than two entities to conduct RE through the following three methods.

#### NetworkX

NetworkX (https://networkx.org) was used to select the main verb with the shortest distance between two entities in a sentence. NetworkX is a Python package for the creation, manipulation, and study of the structure, dynamics, and functions of complex networks, which can compute the shortest paths and path lengths between nodes in the graph.

#### OpenIE

Open Information Extraction (OpenIE) was introduced to extract open-domain relation triples with no schema input for relations in advance. OpenIE has been a critical NLP method for extracting structured relational tuples (subject, relation, object) from unstructured text independently^28^. In addition, OpenIE can express the complete relationship from a sentence to the greatest extent. For example, OpenIE can annotate the relationship as a phrase “be potential targets for” rather than a single verb “be”, which may lose or obscure relational information.

#### AllenNLP

The DL-based method AllenNLP was applied for enriching the relation library. AllenNLP is a complete platform for solving natural language processing tasks in PyTorch, and has developed state-of-the-art deep learning models on a wide variety of linguistic tasks^29^.

### Human aging and longevity biomarkers identification

We further identified human aging and longevity biomarkers by investigating the characteristics of the relationships between gene, RNA, protein, carbohydrate, lipid, peptide, pharmaceutical preparations, toxin, mutation entities and disease entities. The relationships between the potential human aging and longevity biomarkers and disease entities were divided into three classes as follows:

- **Positive relationship**. Positive relationships like “lead” and “cause” were considered aging-promoting relationships.
- **Association relationship**. Relationships that can indicate an association like “associated” and “related” were considered aging-promoting relationships.
- **Negative relationship**. Negative relationships like “prevent” and “ameliorate” were considered longevity-promoting relationships.

## Data Records

The dataset is available at Figshare^30^. HALD includes seven sets of files in JSON and CSV formats: (1) The “Literature_Info.json” file containing the human aging and longevity-related literature information about PMID, title (TI), abstract (AB), IF, IF5, author (AU), full author (FAU), affiliation (AD), publication type (PT), date of publication (DP), place of publication (PL), journal title (JT), journal title abbreviation (TA), and source(SO). (2) The “Entity_Info.json” file containing the information of the entities appearing in the literature about entity, type, official full name, PMID, sentence, number of articles, JT, TA, IF, IF5, year, date, alias names, description, url, mutation position, mutation alleles, MeSH ID, relation, external links, aging biomarker, and longevity biomarker. (3) The “Relation_Info.json” file containing the triples information about source entity, relationship, target entity, method, sentence, source, target, source type, target type, PMID, DP, date, TI, TA, IF, and IF5. (4) The “Aging_Biomarkers.json” file containing the aging biomarkers information about source entity, relationship, target entity, sentence, source, target, source type, target type, PMID, DP, date, TI, TA, IF, and IF5. (5) The “Longevity_Biomarkers.json” file containing the longevity biomarkers information about source entity, relationship, target entity, sentence, source, target, source type, target type, PMID, DP, date, TI, TA, IF, and IF5. (6) The “Entities.csv” file containing the entities information for Neo4j. (7) The “Roles.csv” file containing the relations information for Neo4j. The details are presented in Table 2.

**Table 2:** Dataset details.

File	Objects	Articles	Variables	Short Description
Literature_Info.json	339,145	339,145	PMID, title (TI), abstract (AB), IF (Journal Impact Factor), IF5 (Five-year Journal Impact Factors), author (AU), full author (FAU), affiliation (AD), publication type (PT), date of publication (DP), place of publication (PL), journal title (JT), journal title abbreviation (TA), and source(SO).	JSON file containing the information of human aging and longevity-related literature with abstracts
Entity_Info.json	12,227	181,924	entity, type, official full name, PMID, sentence, number of articles, JT, TA, IF, IF5, years, alias names, description, url, mutation position, mutation alleles, MeSH ID, relation, external links, aging biomarker, and longevity biomarker.	JSON file containing the information of the entities appearing in the literature
Relation_Info.json	115,522	50,191	source entity, relationship, target entity, method, sentence, source, target, source type, target type, PMID, DP, date, TI, TA, IF, and IF5.	JSON file containing the triples information
Aging_Biomarkers.json	1,855	1,502	source entity, relationship, target entity, sentence, source, target, source type, target type, PMID, DP, date, TI, TA, IF, and IF5.	JSON file containing the aging biomarkers information
Longevity_Biomarkers.json	525	494	source entity, relationship, target entity, sentence, source, target, source type, target type, PMID, DP, date, TI, TA, IF, and IF5.	JSON file containing the longevity biomarkers information
Entities.csv	6,906	50,191	ID, name, type, frequency, label	CSV file containing the entities information for Neo4j
Roles.csv	115,514	50,191	start_ID, end_ID, relation, weight, method, type	CSV file containing the relations information for Neo4j

## Technical Validation

### Literature selection

After retrieving the literature, we selected literature with available abstracts in PubMed. While using PubTator, we selected literature with at least one identifier of species equaling “9606” representing *Homo sapiens*. Abstracts were then split into sentences by NLTK^31^.

### Entities normalization

The process of entity normalization is to convert 10 types of entities into corresponding unified standard formats according to the constructed dictionaries. Gene and RNA entities were all converted into approved symbols provided by HGNC, and linked to NCBI gene dababase and HGNC. Carbohydrate, peptide, lipid, protein, pharmaceutical preparations, toxin, and disease entities were all converted into MeSH concepts and terms, and linked to MeSH through a Mesh unique ID. Mutation entities were also linked to dbSNP.

### Main verbs formation

The active or passive form of the verb directly affects the directionality of the relationship, so we performed part-of-speech formation for the main verbs in the sentence. We conducted word segmentation, part-of-speech tagging, and dependency parsing on the sentences with relations. We first removed headings before a colon (such as “INTRODUCTION:”, “METHOD:”, “RESULT:”, and “CONCLUSION:”) in the sentences as they affected the dependency parsing for main verbs formation. Stanza is an NLP library based on PyTorch’s DL framework and neural network algorithms, providing many NLP functions like lemmatization, part-of-speech tagging, NER, and syntactic analysis^32^. Genia is one of the biomedical treebanks for better performing biomedical syntactic analysis from biomedical texts^33^. Therefore, we used the Genia tool provided by Stanza to perform word segmentation, part-of-speech tagging, and dependency syntax analysis on these sentences. Word segmentation was to divide sentences into individual lexical units, while part-of-speech tagging marked the parts of speech of verbs. Verb part-of-speech taggings included verb base (VB), verb past tense (VBD), verb present participle (VBG), verb past participle (VBN), verb non-3rd person singular present (VBP), and verb 3rd person singular present (VBZ). Since the verbs in VB, VBD, VBG, VBP, and VBZ tenses must express the active relationship, we directly transformed them into the original form of the verb to represent the active form of the main verb. However, verbs whose part of speech was VBN may express either an active relationship or a passive relationship, and it was necessary to further study the grammatical relationship between different words through dependency syntactic analysis. A relationship is passive if the past participle of the verb is preceded by the verb “be” or its conjugations, or as a complement to modify the subject or object in the sentence. After the above steps, the main verbs were all transformed into the original form expressing the active relationship and the past participle form expressing the passive relationship, so that they could accurately reflect the directionality of the relationship.

### Negation detection

The presence of negation greatly affects the meaning of sentences, so negative relations were detected and filtered out. Part-of-speech tagging also marked the parts of speech of adverbs including adverbs (RB), adverb comparative (RBR), and adverb superlative (RBS). Negative words (such as “no”, “not”, “never”, “hardly”, “barely”, “scarcely”, “rarely”, “few”, “little”, “seldom”, “neither”, and “nor”) whose part of speech was adverb were detected and such relations were excluded while conducting RE.

### Framework of KG

Generally, resource description framework (RDF) and graph database are two main storage forms of KG. RDF is convenient for designers to publish and share data, while graph dababase provides a user-friendly interface to browse data. Thus, we developed the graph database-based HALD to explore the human aging and longevity-related KG. The front end was built with React (https://react.dev/) and Elasticsearch (https://www.elastic.co/) was used to realize a real-time search and management. We employed Neo4j (https://neo4j.com/) to offer an intuitive network demonstration of the entities and relations knowledge. All analyses in this study were done inside JupyterLab (https://jupyter.org/) notebooks with the Python kernel. Automatic updates would be executed monthly to keep the KG up-to-date.

### Evaluation of HALD

Since genes make up the largest proportion of entities in HALD, we compared genes documented in HALD with those manually curated in GenAge^9^, LongevityMap^9^, Aging Atlas^11^ and AgingBank^12^. GenAge collects genes that are possibly related to human aging^9^. LongevityMap is a database of human genetic variants associated with longevity^9^. Aging Atlas provides a section of aging-related genes that can be regarded as aging biomarkers^11^. AgingBank is a manually curated knowledgebase of aging offering a table that can be filtered by “Pro/Anti” tags to classify the genes into aging and longevity groups^12^.

For aging-related genes, we compared HALD with the other three databases GenAge, Aging Atlas and AgingBank (Pro) (Fig. 2c). HALD contained the most aging-related genes, which provided a rich resource for aging biomarkers. 54.2%, 47.6%, and 58.2% of the aging-related genes in GenAge, Aging Atlas, and AgingBank (Pro) overlapped with aging-related genes in HALD respectively (Table 3). For longevity-related genes, we compared HALD with the other two databases LongevityMap and AgingBank (Anti) (Fig. 2d). HALD collected 396 longevity-related genes compared with 766 in LongevityMap and 99 in AgingBank (Anti). 10.4% and 27.3% of the longevity-related genes in LongevityMap and AgingBank (Anti) overlapped with genes in HALD (Table 3). It may be because HALD only extracted longevity-related entities with negative relationships mentioned in the title and abstract, while LongevityMap and AgingBank primarily curated entities from the full text manually. Furthermore, the top 10 aging and longevity-related genes in HALD were used to compare with the other databases (Table 3). These genes in HALD had high coverage with all the other databases except AgingBank (Pro). Considering that limited words extracted from a sentence might not accurately divide the entities into aging and longevity-related biomarkers, we also considered combining the biomarkers of aging and longevity in HALD together and compare them with the other five databases. We found that approximately 50% of genes overlapped except LongevityMap, and the proportion of the top ten overlapping genes has shown a significant increase in AgingBank.

**Table 3:** Comparisons of gene count between GenAge, LongevityMap, Aging Atlas, AgingBank and HALD.

HALD	Other databases	Genes in HALD	Genes in other databases	Overlapping genes	Percentage in other databases	Top 10 overlapping genes
Aging biomarkers	GenAge	1353	306	166	54.2%	7
	Aging Atlas	1353	500	238	47.6%	8
	AgingBank (Pro)	1353	55	32	58.2%	3
Longevity biomarkers	LongevityMap	396	766	80	10.4%	7
	AgingBank (Anti)	396	99	27	27.3%	7
Aging and longevity biomarkers	GenAge	1473	306	173	56.5%	7
	LongevityMap	1473	766	235	30.7%	7
	Aging Atlas	1473	500	247	49.4%	8
	AgingBank	1473	144	77	53.5%	7

**Fig. 3:**
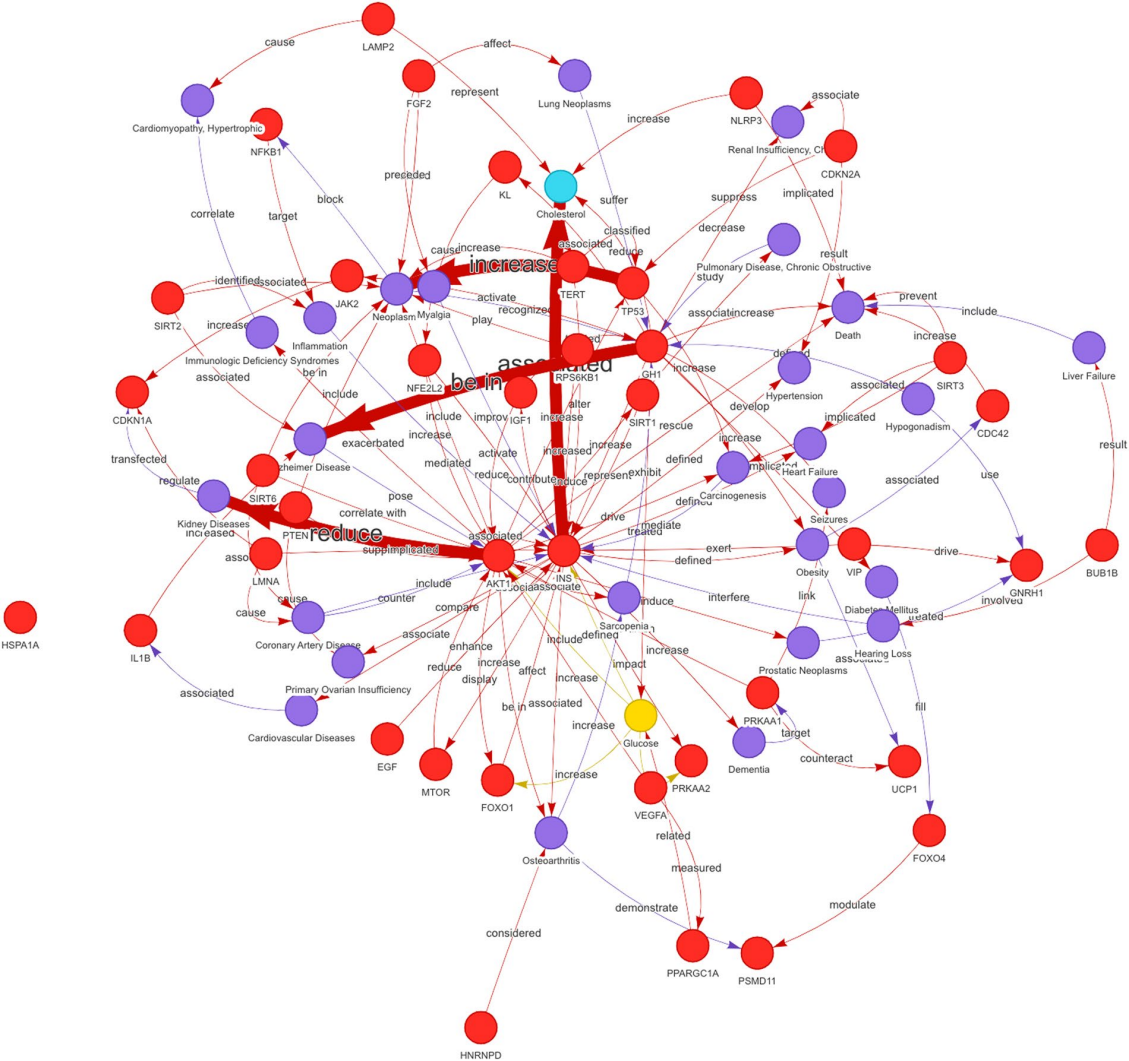
The network of entities. The entities mentioned by López-Otn *et al*.^1^ were used for predicting the relationships.

In addition to comparing with known databases on human aging and longevity, we have also chosen two related studies for further evaluation^1,34^. López-Otn *et al*. proposed nine tentative hallmarks of aging to represent common factors of aging in different organisms, particularly in mammalian organisms^1^. We filtered all biomedical entities related to mammals from the literature and compared them with the entities collected in HALD. Apart from the telomere attrition feature, the other eight aging features all contained a certain number of entities. This is mainly because entities of telomere attrition tend to relate to the length of telomeres. In total, we extracted 37 different entities from the articles, all of which were found to match the entities in HALD after normalization (Table S1). We predicted the relationships of these entities (Fig. 3) and found that all the entities were connected through the network except HSPA1A. Furthermore, we compared these entities with the biomarkers for aging and longevity identified in HALD and found that 33 out of 37 matched, except for HP1*α*^35^, Hsp72^36^, PGC-1*α*^37,38^, and PGC-1*β*^38^. We checked these articles manually and discovered that they were all related to mouse organisms and may not have been extensively studied in the context of human aging and longevity.

We also downloaded 258 potential aging biomarkers provided by the TAME Trial investigators^34^. Measurements like height and CD4/CD8 T cell ratio have less responsiveness to intervention, while biological small molecules typically exert their biological functions by participating in the formation and regulation of macromolecules. Therefore, we filtered out the entities under the situations of biological measurement, cell, medical measurement, physical measurement, questionnaire, amino acid, nucleotide, and radical. In this way, 108 entities were left and we found that all of them matched the HALD dataset (Table S2). Justice *et al*. also applied four strict selection criteria to TAME and proposed the following ten biomarkers: IL-6, TNFÎ ± -receptor I or II, CRP, GDF15, insulin, IGF1, cystatin C, NT-proBNP, and hemoglobin A1c^34^. HALD not only comprehensively documented these ten entities but also accurately identified them as human aging and longevity biomarkers, which once again demonstrated the reliability of HALD.

## Usage Notes

We designed HALD with four critical components, including (1) 339,918 articles related to human aging and longevity, (2) 10 types of entities (gene, RNA, carbohydrate, peptide, lipid, protein, pharmaceutical preparations, toxin, mutation, and disease), (3) 115,522 relations, and (4) 1,855 aging biomarkers and 525 longevity biomarkers. The presentation of the HALD dataset aimed to provide significant convenience to researchers in the field of human aging and longevity and reduce the workload of further sifting through vast amounts of data. Additionally, HALD predicted biomarkers of aging and longevity from published literature, making it a valuable reference for precision gerontology and geroscience analyses. HALD is publicly available at Figshare^30^, an open scientific data repository. For scientific researchers who want to explore the dataset intuitively, please visit https://bis.zju.edu.cn/hald for user-interactive browsing.

Below we demonstrate two use cases of how HALD may be used by researchers from different fields.

For researchers in the field of molecular biology, their primary concern lies in the biomarkers associated with human aging and longevity. For those without the programming background, directly using the online website can be more convenient. For example, they may want to first explore the genes most studied in the past decade. They can do this by using the website’s Search module, selecting “gene” as the entity type, specifying the year range from 2013 to 2023, and setting the filter mode to “Most”. The page will display information about the top ten genes, which currently are “INS, CRP, IL6, APOE, CD4, TNF, MAPT, ALB, SIRT1, and CD8A.” They can also search for the gene names of interest, and the auto-complete input box will suggest the most relevant entities. After searching, normalized entity names and related information will be displayed, including official full name, alias, summary, external links, and the number of related articles. External links can directly navigate to other databases, including NCBI, GenAge, LongevityMap, Aging Atlas and AgingBank. Furthermore, to the right of each gene label in the first row, there are tags for Network, Aging, and Longevity modules. Clicking on these tags allows them to access the corresponding pages. For detailed information about these top ten genes, they can click on the gene label to navigate to more specific tabular data, which includes details like PMID, source, type, JT, TA, DP, year, IF, IF5, and the sentences in which the entity is mentioned. In the table, each column is filterable, allowing them to easily filter results based on factors such as DP, IF and IF5. Additionally, the table supports the ability to download filtered results.

For clinical doctors, the focus lies in understanding the mechanisms underlying aging and longevity-related diseases, with a particular emphasis on the relationship between biological molecules and diseases. They can navigate to the Network module, filter the target entity type as “disease”, enter “Alzheimer Disease” in the input box, and limit the quantity to 50. Specifically, they can query direct or indirect relationships between nodes by setting the minimum and maximum number of relationships. Direct relationships are manifested as a triple present in a single sentence, while indirect relationships involve two or more sentences and are a form of predicted relationships. After clicking the “Load Network” button, a network graph composed of nodes and edges will be displayed in the lower left corner. When hovering the mouse over a node, a tooltip will display the entity’s name, frequency, and type. When hovering the mouse over an edge, it will show the relationship, weight and method. Weight for all the edges is the sum of the occurrences of that relation in various literature. Clicking on nodes or edges will display corresponding reference information on the right half of the webpage, including the sentence, source, relationship, target, method, relationship, JT, IF, IF5, DP, PMID, and TI. These details also support filtering and downloading based on date, IF, and IF5. Furthermore, the Aging and Longevity modules provide detailed information about the entities predicted as biomarkers of aging and longevity in this dataset, along with filtering and downloading capabilities.

Further data analysis can be expanded by directly downloading the HALD dataset. Users are welcome to contribute data and give suggestions in the Feedback module on the website at any time, by directly filling the form and click the “FEEDBACK” button to submit it. We will promptly check all the feedback, respond via email, and make necessary adjustments as soon as possible.

### Supplementary information

Supplementary Information

## Data Availability

All code used in this paper can be downloaded on GitHub at https://github.com/zexuwu/hald.

## References

[1] López-Otn C, Blasco MA, Partridge L, Serrano M, Kroemer G (2013). The hallmarks of aging. Cell.

[2] Khan SS, Singer BD, Vaughan DE (2017). Molecular and physiological manifestations and measurement of aging in humans. Aging Cell.

[3] Semerciöz-Oduncuoğlu AS, Mitchell SE, Özilgen M, Yilmaz B, Speakman JR (2023). A step toward precision gerontology: lifespan effects of calorie and protein restriction are consistent with predicted impacts on entropy generation. Proc. Natl. Acad. Sci. USA.

[4] Seals DR, Justice JN, LaRocca TJ (2016). Physiological geroscience: targeting function to increase healthspan and achieve optimal longevity. J. Physiol.-London.

[5] Zhao S, Su C, Lu Z, Wang F (2021). Recent advances in biomedical literature mining. Brief. Bioinform..

[6] Wei C-H, Kao H-Y, Lu Z (2013). PubTator: a web-based text mining tool for assisting biocuration. Nucleic Acids Res..

[7] Li P-H (2022). pubmedKB: an interactive web server for exploring biomedical entity relations in the biomedical literature. Nucleic Acids Res..

[8] Kaeberlein M, Jegalian B, McVey M (2002). AGEID: a database of aging genes and interventions. Mech. Ageing Dev..

[9] Tacutu R (2012). Human Ageing Genomic Resources: integrated databases and tools for the biology and genetics of ageing. Nucleic Acids Res..

[10] Hühne R, Thalheim T, Sühnel J (2014). AgeFactDB–the JenAge Ageing Factor Database–towards data integration in ageing research. Nucleic Acids Res..

[11] Consortium AA (2021). Aging Atlas: a multi-omics database for aging biology. Nucleic Acids Res..

[12] Gao Y (2022). AgingBank: a manually curated knowledgebase and high-throughput analysis platform that provides experimentally supported multi-omics data relevant to aging in multiple species. Brief. Bioinform..

[13] Li Z (2021). Aging and age-related diseases: from mechanisms to therapeutic strategies. Biogerontology.

[14] Ji S, Pan S, Cambria E, Marttinen P, Philip SY (2021). A survey on knowledge graphs: representation, acquisition, and applications. IEEE Trans. Neural Netw. Learn. Syst..

[15] Lin, Y., Liu, Z., Sun, M., Liu, Y. & Zhu, X. Learning entity and relation embeddings for knowledge graph completion. In *Proceedings of the AAAI conference on artificial intelligence*, **vol. 29** (2015).

[16] Cock PJ (2009). Biopython: freely available python tools for computational molecular biology and bioinformatics. Bioinformatics.

[17] Manning, C. *et al*. The Stanford CoreNLP natural language processing toolkit. In *Proceedings of 52nd Annual Meeting of the Association for Computational Linguistics: System Demonstrations*, 55–60 (2014).

[18] Neumann, M., King, D., Beltagy, I. & Ammar, W. ScispaCy: Fast and robust models for biomedical natural language processing. In *Proceedings of the 18th BioNLP Workshop and Shared Task*, 319–327 (Association for Computational Linguistics, Florence, Italy, 2019).

[19] Kim D (2019). A neural named entity recognition and multi-type normalization tool for biomedical text mining. IEEE Access.

[20] Wei, C.-H., Kao, H.-Y. & Lu, Z. GNormPlus: an integrative approach for tagging genes, gene families, and protein domains. *Biomed Res. Int.* **2015**, 918710 (2015). 10.1155/2015/918710 PMC4561873 26380306

[21] Wei C-H (2018). tmVar 2.0: integrating genomic variant information from literature with dbSNP and ClinVar for precision medicine. Bioinformatics.

[22] Leaman R, Islamaj Doğan R, Lu Z (2013). DNorm: disease name normalization with pairwise learning to rank. Bioinformatics.

[23] Povey S (2001). The HUGO gene nomenclature committee (HGNC). Hum. Genet..

[24] Lipscomb CE (2000). Medical subject headings (MeSH). Bull. Med. Libr. Assoc..

[25] Sherry ST (2001). dbSNP: the NCBI database of genetic variation. Nucleic Acids Res..

[26] Honnibal M, Montani I, Van Landeghem S, Boyd A (2020). Zenodo.

[27] Lee J (2020). BioBERT: a pre-trained biomedical language representation model for biomedical text mining. Bioinformatics.

[28] Dong, K., Yilin, Z., Sun, A., Kim, J. J. & Li, X. DocOIE: a document-level context-aware dataset for OpenIE. In *Findings of the Association for Computational Linguistics: ACL-IJCNLP 2021*, 2377–2389 (2021).

[29] Gardner, M. *et al*. AllenNLP: a deep semantic natural language processing platform. In *Proceedings of Workshop for NLP Open Source Software (NLP-OSS)* (Association for Computational Linguistics, 2018).

[30] Wu Z (2023). figshare.

[31] Bird, S., Klein, E. & Loper, E. *Natural Language Processing with Python: Analyzing Text with the Natural Language Toolkit* (O’Reilly Media, 2009).

[32] Qi, P., Zhang, Y., Zhang, Y., Bolton, J. & Manning, C. D. Stanza: A python natural language processing toolkit for many human languages. In *Proceedings of the 58th Annual Meeting of the Association for Computational Linguistics: System Demonstrations*, 101–108 (2020).

[33] Kim J-D, Ohta T, Tateisi Y, Tsujii J (2003). GENIA corpus–a semantically annotated corpus for bio-textmining. Bioinformatics.

[34] Justice JN (2018). A framework for selection of blood-based biomarkers for geroscience-guided clinical trials: report from the TAME Biomarkers Workgroup. GeroScience.

[35] Schotta G (2004). A silencing pathway to induce H3-K9 and H4-K20 trimethylation at constitutive heterochromatin. Genes Dev..

[36] Gehrig SM (2012). Hsp72 preserves muscle function and slows progression of severe muscular dystrophy. Nature.

[37] Rodgers JT (2005). Nutrient control of glucose homeostasis through a complex of PGC-1*α* and SIRT1. Nature.

[38] Sahin E, DePinho RA (2012). Axis of ageing: telomeres, p53 and mitochondria. Nat. Rev. Mol. Cell Biol..

