# Supplementary material for: HALD, a human aging and longevity knowledge graph for precision gerontology and geroscience analyses
Source: Sci Data. 2023 Dec 1;10:851. doi: 10.1038/s41597-023-02781-0 (PMC10692171; doi:10.1038/s41597-023-02781-0)
Supplement: Supplementary file 1 — Supplementary Information [file 41597_2023_2781_MOESM1_ESM.pdf]

Supplementary Information

HALD, a human aging and longevity knowledge graph for precision gerontology and geroscience analyses

Zexu Wu[1,†], Cong Feng[1,2,†], Yanshi Hu[1], Yincong Zhou[1,3], Sida Li[1], Shilong Zhang[1], Yueming Hu[1], Yuhao Chen[1], Haoyu Chao[1], Qingyang Ni[1], and Ming Chen[1,2,3,*]

[1]Department of Bioinformatics, College of Life Sciences, Zhejiang University, Hangzhou, 310058, China
[2]Bioinformatics Center, The First Affiliated Hospital, School of Medicine, Zhejiang University, Hangzhou, 310058, China
[3]Joint Research Centre for Engineering Biology, Zhejiang University-University of Edinburgh Institute, Zhejiang University, Haining, 314400, China
[*]corresponding author(s): Ming Chen (mchen@zju.edu.cn)
[†]these authors contributed equally to this work

Table S1. Comparisons of entities between the hallmarks of aging and HALD.
Table S2. Comparisons of entities between TAME and HALD.

Table S1. Comparisons of entities between the hallmarks of aging and HALD

Hallmarks	Subsection	Entity	Entities in HALD	Aging or Longevity Biomarkers
**Genomic Instability**	**Nuclear DNA**	BubR1	BUB1B	Aging
	**Nuclear Architecture**	p53	TP53	Aging & Longevity
		NF-κB	NFKB1	Aging & Longevity
		LMNA	LMNA	Aging
**Epigenetic Alterations**	**Histone Modifications**	insulin	INS	Aging & Longevity
		IGF-1	IGF1	Aging & Longevity
		SIRT1	SIRT1	Aging & Longevity
		SIRT6	SIRT6	Aging & Longevity
		SIRT3	SIRT3	Aging & Longevity
	**Chromatin Remodeling**	HP1α	CBX5	-
	**Reversion of Epigenetic Changes**	SIRT1	SIRT1	Aging & Longevity
**Loss of Proteostasis**	**Chaperone-Mediated Protein Folding and Stability**	HSF-1	HSF1	Aging
		SIRT1	SIRT1	Aging & Longevity
		Hsp72	HSPA1B | HSPA1A	-
	**Proteolytic Systems**	LAMP2a	LAMP2	Aging
		mTOR	MTOR	Aging & Longevity
		S6K1	RPS6KB1	Aging
		EGF	EGF	Aging & Longevity
		RPN-6	PSMD11	Aging
**Deregulated Nutrient Sensing**	**The Insulin- and IGF-1-Signaling Pathway**	insulin	INS	Aging & Longevity
		IGF-1	IGF1	Aging & Longevity
		FOXO1	FOXO1	Aging & Longevity
		PTEN	PTEN	Aging & Longevity
		GH	GH1	Aging & Longevity
		AKT	AKT1	Aging & Longevity
	**Other Nutrient-Sensing Systems: mTOR, AMPK, and Sirtuins**	mTOR	MTOR	Aging & Longevity
		AMPK	PRKAA1 | PRKAA2	Aging & Longevity
		SIRT1	SIRT1	Aging & Longevity
		PGC-1α	PPARGC1A	-
**Mitochondrial Dysfunction**	**Mitochondrial Integrity and Biogenesis**	PGC-1α	PPARGC1A	-
		PGC-1β	PPARGC1B	-
		SIRT1	SIRT1	Aging & Longevity
		SIRT3	SIRT3	Aging & Longevity
	**Mitohormesis**	AMPK	PRKAA1 | PRKAA2	Aging & Longevity
		NRF2	NFE2L2	Aging & Longevity
		PGC-1α	PPARGC1A	-
		UCP1	UCP1	Aging

		p16	CDKN2A	Aging & Longevity
**Cellular Senescence**	**The INK4a/ARF Locus and p53**	p19	CDKN2A	Aging & Longevity
		p53	TP53	Aging & Longevity
		p16	CDKN2A	Aging & Longevity
		p21	CDKN1A	Aging
**Stem Cell Exhaustion**		FGF2	FGF2	Aging
		mTORC1	MTOR	Aging & Longevity
		CDC42	CDC42	Aging
		NF-κB	NFKB1	Aging & Longevity
		NLRP3	NLRP3	Aging & Longevity
		IL-1β	IL1B	Aging & Longevity
		GnRH	GNRH1	Aging & Longevity
**Altered Intercellular Communication**	**Inflammation**	AUF1	HNRNPD	Aging
		TERT	TERT	Aging & Longevity
		SIRT1	SIRT1	Aging & Longevity
		SIRT2	SIRT2	Aging & Longevity
		SIRT6	SIRT6	Aging & Longevity

Table S2. Comparisons of entities between TAME and HALD

TAME	HALD	Aging or Longevity Biomarkers
Adiponectin	ADIPOR1 | ADIPOR2	-
Akt	AKT1 | AKT2 | AKT3 | AKT1S1	Aging & Longevity
Alanine aminotransferase (ALT)	GPT	Aging & Longevity
Albumin	ALB	Aging
Alkaline phosphatase, bone-specific	ALPI | ALPL	Aging
AMPK activation	PRKAA1 | PRKAA2 | PRKAB1	Aging & Longevity
Amyloid A (acute phase protein)	SAA1 | SAA4	-
Aortic valve calcification	Vascular Calcification	Aging & Longevity
APOE genotype	APOE	Aging & Longevity
Apolipoproteins (ApoA1, ApoB)	APOA1 | APOB	Aging
Aspartate aminotransferase (AST)	GOT1	-
Atg (autophagy)	ATG4A | ATG2B | ATG4C | ATG12 | ATG5 | ATG7 | ATG16L1 | ATG10 | ATG9B |ATG13 | ATG4D | ATG3 | ATG4B	Aging & Longevity
Beta 2 microglobulin	B2M	-
Beta-amyloid	APP | APLP2 | APBB1 | APBB2 | APBA1 | APBA2	Aging
Bicarbonate	SLC4A4	-
C- Reactive Protein (CRP)	CRP	Aging & Longevity
Caveolin-1	CAV1	Aging
CCL11 (eotaxin)	CCL11	Aging
Chloride	Cholesterol	Aging & Longevity
Cholesterol, HDL	Cholesterol	Aging & Longevity
Cholesterol, LDL	Cholesterol	Aging & Longevity
Cholesterol, Total	Cholesterol	Aging & Longevity
Cholesterol, Total / HDL ratio	Cholesterol	Aging & Longevity
Collagen turnover	Collagen Diseases	Aging & Longevity
Collagen, procollagen type1	COL1A1	Aging
Cystatin C	CST3	Aging
Eotaxin (CCL11)	CCL11	Aging
Ext matrix remodeling (MMP9)	MMP9	Aging
Ferritin	FTL | FTH1	-
Fibrinogen	FGB | FGG	Aging
Fibroblast growth factor 23	FGF23	Aging
Folate	FOLH1 | FOLR2	Aging
Folate, RBC	FOLH1 | FOLR2	Aging
Free fatty acids	Fatty Acids, Nonesterified	Aging
g-H2A.X	H2AX	-
Gamma-glutamyl transpeptidase	GGT1	-
Glucose, fasting	Glucose	Aging & Longevity
Glutathione, glutat. reductase/peroxidase	GSR | GPX1	Aging

Glycated hemoglobin (HbA1c)	HBA1	Aging
Growth Differentiating Factor 11/8	GDF11 | GDF8	Aging & Longevity
Growth Differentiating Factor 15	GDF15	Aging & Longevity
Growth hormone	Growth Hormone	-
Heat shock proteins	DNAJB6 | HSPA9 | HSPB2 | HSPA1A | HSPA1B | HSPB1 | HSPD1 | HSPA8 | HSPA5 | HSPB8 | DNAJB9 | HSP90B1 | HSPA2 | DNAJA3 | DNAJC19 | HSPA14 | HSPA13 | HSPA6 | HSPA6 | DNAJB1 | DNAJC3 | DNAJC7 | DNAJC6 | DNAJC5 | HSPA1L | DNAJC5B | HSP90AA1 | HSP90AB1 |	Aging & Longevity
Hemoglobin	HBB | HBA1 | AHSP | HBG2	Aging
ICAM-1 or 2	ICAM1	Aging
Insulin-like growth factor -1 (IGF-1)	IGF1	Aging & Longevity
Insulin-like growth factor BP-1	IGFBP1	Aging
Insulin-like growth factor BP3	IGFBP3	Aging & Longevity
Insulin, fasting	INS	Aging & Longevity
Interferon gamma (IFN-γ)	IFNG	Aging
Interleukin 1β (Il-1β)	IL1B	Aging & Longevity
Interleukin-2 (Il-2)	IL2	Aging & Longevity
Interleukin 8 (IL-8)	CXCL8	Aging & Longevity
Interleukin-6 (IL-6), basal	IL6	Aging & Longevity
Interleukin-6 (IL-6), post-stimulation	IL6	Aging & Longevity
Isoprostanes (iPF2alpha - III, VI)	Isoprostanes	Aging
Klotho	KL	Aging & Longevity
Lactate dehydrogenase, LDH	LDHA | LDHB | LDHC	Aging
Leptin	LEP	Aging & Longevity
Metallothioneins (MTs)	MT1B | MT1G | MT2A | MT1A | MT1X | MT1H	Aging
Monocyte chemoattractant protein 1	CCL2	Aging
Natriuretic peptides, NT-pro BNP	NPPA | NPPB | NPPC	Aging & Longevity
Nicotinamide phosphoribosyltransferase (visfatin)	NAMPT	Aging & Longevity
Norepinephrine	SLC6A2	-
Oxidized low-density lipoprotein	OLR1	-
p16INK4a	CDKN2A	Aging & Longevity
p19ARF	CDKN2A	Aging & Longevity
p53, p21	TP53	Aging & Longevity
p62 (autophagy)	NUP62 | SQSTM1	Aging
PARP-1	PARP1	-
Pentraxin-3	PTX3	Aging
PGC-1a	PPARGC1A	-
Plasminogen activator inhibitor1	SERPINE1	Aging
Proinsulin	INS	Aging & Longevity
Prostate specific antigen	KLK3	Aging & Longevity
Sex hormone-binding globulin (SHBG)	SHBG	Aging & Longevity
Sex hormones (LH, FSH)	LHB	-

Sirtuin-1, sirtuin-2	SIRT1 | SIRT2	Aging & Longevity
Superoxide dismutase	SOD1 | SOD2 | SOD3	Aging & Longevity
T cells, Senescent memory CD4	CD4	Aging & Longevity
Telomerase; reverse transcriptase	TERT	Aging & Longevity
Thioredoxin reductase-1	TXNRD1	-
Transferrin receptor (Transf. R)	TFRC	Aging
Transforming growth factor β (TGF β)	TGFB1 | TGFB2 | TGFB3	-
Triglycedride	Triglycerides	Aging & Longevity
Troponin	TNNT1 | TNNT2 | TNNT3 | TNNI2 | TNNI3	-
Tumor Necrosis Factor α (TNFα)	TNF	Aging & Longevity
Tumor Necrosis Factor post-stim.	TNF	Aging & Longevity
Tumor Necrosis Factor Receptors	TNFRSF1B	Longevity
Vascular endothelial growth factor	VEGFA | VEGFB | VEGFC | VEGFD	Aging & Longevity
von Willebrand factor	VWF	Aging & Longevity
S100beta	S100B	Aging
Lipoprotein-associated phospholipase A2 (Lp-PLA2)	PLA2G7	-
Vascular cell adhesion molecule (VCAM)	VCAM1	Aging
Copeptin (c-terminal provasopressin)	AVP	Aging
Cardiac troponin	TNNT2 | TNNT3	-
Creatine-Kinase-MB	CKB	-
Myeloperoxidase	MPO	Aging & Longevity
Heart-type fatty acid binding protein	FABP3	Aging & Longevity
Endothelin-1 (ET1) or C-terminal pro-Endothelin-1 (CTproET1)	EDN1	Aging
Supressor of tumorgenecity 2 (ST2)	IL1RL1	-
Galectin-3 (gal-3)	LGALS3	-
Tissue inhibitors of metalloproteinases (TIMPs)	TIMP1 | TIMP2 | TIMP3 | TIMP4	Aging & Longevity
plasma T-tau	MAPT	Aging & Longevity
HER-2 (ERBB2) overexpression / amplification	ERBB2	Aging & Longevity
α-fetoprotein (AFP)	AFP	Aging
Human chorionic gonadotropin-β (β- hGC)	CGB5	-
Thyroglobulin (TG)	TG	Aging
